# 细胞周期检测点激酶与肺癌耐药研究进展

**DOI:** 10.3779/j.issn.1009-3419.2021.101.09

**Published:** 2021-04-20

**Authors:** 芷茵 柯, 爱玲 梁, 勇军 刘

**Affiliations:** 1 523808 东莞，广东省医学分子诊断重点实验室 Medical Molecular Diagnostics Key Laboratory of Guangdong, Dongguan 523808, China; 2 523808 东莞，广东医科大学基础医学院生物化学与分子生物学教研室 Department of Biochemistry and Molecular Biology Guangdong Medical University, Dongguan 523808, China; 3 523808 东莞，广东医科大学医学技术学院临床生物化学教研室 Department of Clinical Laboratory Biochemistry in Guangdong Medical University, Dongguan 523808, China

**Keywords:** 细胞周期检测点激酶, 肺肿瘤, 耐药, Cell cycle checkpoint kinase, Lung neoplasms, Drug resistance

## Abstract

肺癌是最常见的诊断癌症和癌症死亡的主要原因。虽然化疗、放疗、靶向治疗等治疗手段取得了重大进展，但获得性耐药的出现阻碍了临床治疗的疗效。研究表明肿瘤是一类细胞周期调控机制破坏的疾病，其中细胞周期检测点激酶（checkpoint kinase, Chk）发挥着核心的作用，而Chk1和Chk2是检测点中非常重要的蛋白激酶。近年来发现，调控Chk1和Chk2对肺癌的临床治疗和耐药机制有十分重要的意义。本文就细胞周期检测点激酶与肺癌耐药机制进行综述，阐述有效的肺癌治疗靶点和方法。

## Abstract (en)

Lung cancer is the most commonly diagnosed cancer and the leading cause of cancer death. Although great progress has been made in chemotherapy, radiotherapy and targeted therapy, the emergence of acquired drug resistance hinders the efficacy of clinical treatment. Studies have shown that tumor is a class of diseases with damaged cell cycle regulation mechanism, in which checkpoint kinase (Chk) plays a core role, Chk1 and Chk2 are very important protein kinases in the checkpoint. In recent years, it has been found that the regulation of Chk1 and Chk2 plays an important role in the clinical treatment and drug resistance mechanism of lung cancer. This article reviews the mechanism of cell cycle checkpoint kinase and drug resistance of lung cancer, and expounds the effective therapeutic targets and methods of lung cancer.

肺癌是最常见的诊断癌症和癌症死亡的主要原因，其中非小细胞肺癌（non-small cell lung cancer, NSCLC）约占肺癌的85%^[1]^。在男性中，肺癌是最常见的癌症（占癌症病例总数的14.5%），也是癌症死亡的主要原因（占癌症死亡总数的22.0%）。在女性中，肺癌是仅次于乳腺癌的最常见癌症死亡原因（占癌症死亡总数的13.8%）^[2]^。与大多数国家相比，中国的肺癌死亡率相对较高。据预测，至2030年中国的肺癌死亡率可能增加约40%^[3]^。近年来，虽然手术、化疗、放疗等常规治疗手段取得了很大进展，但肺癌整体5年生存率仅为15%。靶向治疗的出现给肺癌的治疗带来了革命性的变化，但获得性耐药的出现不可避免地阻碍了治疗的效果^[4]^。

肿瘤是一类细胞周期调控机制破坏的疾病，在细胞周期进程的整套监督体系中，细胞周期检测点发挥着核心的作用。Chk1和Chk2是细胞周期检测点中非常重要的蛋白激酶，它们通过信号传导和放大，调节下游靶蛋白活性与表达，使细胞周期出现阻滞^[5]^。化疗药物耐药是导致肿瘤患者复发、治疗难度加大、治疗失败和预后不良的重要原因，而DNA损伤应答（DNA damage response, DDR）是影响肿瘤化疗耐药的一个重要途径，靶向DDR成分中DNA修复相关激酶或检测点可以提供新的解决方案。近年来，调控DDR这一过程中，Chk1及Chk2分子引起了国内外学者的广泛关注，人们期望引发有丝分裂灾难从而促进细胞死亡，而Chk1和Chk2正是癌症治疗中有前景的靶点^[6]^。目前已有Chk抑制剂应用于临床，并在肺癌中取得了成效，其通过诱导细胞凋亡，使细胞周期阻滞，从而逆转细胞周期失调引起的获得性耐药。

## 1 Chk概述

Chk1，最初在1993年发现，是一种丝氨酸/苏氨酸（Ser/Thr）蛋白激酶，在裂殖酵母菌的DNA损伤反应中控制着G_2_期/M期的转变。人类*Chk1*基因位于染色体11q22-23，由476个氨基酸残基组成，在进化上高度保守。同时，Chk1也是脊椎动物细胞中DNA损伤和复制检查点的主调节因子，在调节细胞周期和参与DNA损伤修复中发挥着关键作用，并且可以影响细胞的存活和凋亡^[7]^。

Chk2，由N端SQ/TQ簇结构域（SQ/TQ cluster domain, SCD）、中心叉头相关结构域（forkhead associated domain, FHA）和C端丝氨酸/苏氨酸激酶结构域（kinase domain, KD）构成。人类*Chk2*基因位于染色体22q12.1，由543个氨基酸残基组成。Chk2是DNA损伤反应的关键调节因子和肿瘤抑制因子，也是细胞存活和各种病理生理过程的重要监督者，参与促进细胞周期阻滞、凋亡和DNA修复^[8, 9]^。

人类基因组DNA常常受到多种因素的威胁，包括来自内部和外部因素的影响，如复制应激、电离辐射、X射线暴露和化疗药物等。这些因素的共同作用，使得DNA损伤在哺乳动物细胞中反复发生，触发DDR^[10]^。正常细胞有完整的G_1_期、S期和G_2_期/M期检测点，细胞的存活依赖于激活检测点，引起细胞周期阻滞而启动DNA损伤修复系统，而其中参与DDR途径的两种关键检测点激酶，分别为Chk1和Chk2^[6]^。Chk1使细胞周期停滞在S期或G_2_期/M期，阻止DNA受损的细胞进入有丝分裂^[11]^；Chk2参与细胞周期G_1_期，S期或G_2_期/M期的阻滞，促进细胞对损伤进行修复，维持基因组的稳定。P53在控制G_1_期检测点方面起着核心作用，而大多数癌症由于*p53*的缺乏导致G_1_期检测点调控缺陷，迫使癌细胞在DNA损伤后高度依赖于Chk1激活的S期和G_2_期/M期检测点进行修复^[12]^。

当发生DNA断裂时，Chk1和Chk2分别由共济失调毛细血管扩张症和Rad3相关激酶（ataxia telangiectasia and Rad3 related kinase, ATR）和共济失调毛细血管扩张症突变激酶（ataxia telangiectasia mutated kinase, ATM）磷酸化激活，形成两条经典的信号转导通路，即ATR-Chk1-细胞分裂周期蛋白25（cell division cyclin 25, Cdc25）和ATM-Chk2-Cdc25信号通路^[13, 14]^。当DNA发生单链断裂（single-strand breaks, SSBs）时，ATR-Chk1-Cdc25信号通路被激活，启动DNA损伤修复系统。上游的ATR将DNA的受损信号通过中介因子传递至Chk1，Chk1定位聚集在DNA损伤位点，并被ATR在Ser317和Ser345这两个位点磷酸化而被激活，然后Chk1磷酸化抑制其下游的细胞周期蛋白依赖性激酶（cyclin-dependent kinase, CDK）激活物Cdc25A，使Cdc25A加速泛素化并降解，从而抑制CDK的激活，促使细胞周期在S期/G_2_期或G_2_期/M期阻滞^[15, 16]^。当DNA发生双链断裂（double-strand breaks, DSBs）时，ATM-Chk2-Cdc25信号通路被激活。*Chk2*基因突变后，其编码的激酶会丧失活性，无法修复损伤的DNA，受损伤的DNA不断复制，产生大量异常细胞，进而导致癌变。Chk2作为一个高度保守的蛋白激酶，在修复过程中起着重要的作用^[17]^。其在复制缺陷、电离辐射及靶向DNA的药物等时被激活，活化的Chk2可以磷酸化并且稳定20多种底物蛋白，包括细胞周期相关转录因子1（E2F transcription factor 1, E2F-1）、乳腺癌易感基因1（breast cancer susceptibility gene 1, BRCA1）、P53、Cdc25A、Cdc25C等，使细胞周期发生阻滞，同时激活修复基因的转录，促进细胞对损伤的修复^[18]^。

## 2 Chk与肿瘤耐药

大量研究表明，Chk1和Chk2在正常细胞中均有一定程度的表达，但在肿瘤细胞中却表现为表达水平的异常。目前认为，Chk1在肿瘤细胞或组织中高表达，对肿瘤的增殖和生存发挥重要作用；而Chk2在肿瘤中的表达尚不一致，其缺失或过表达都可能会影响肿瘤的发生发展。癌细胞DNA损伤修复能力的增强是获得性化疗耐药的机制之一。随着Chk1抑制剂和Chk1/Chk2双重抑制剂在临床试验中的应用，Chk1和Chk2在肿瘤耐药中的重要地位正逐步得到认可。

### 2.1 Chk1与肿瘤耐药

目前已经发现Chk1在多种人类肿瘤中过表达，包括乳腺癌、结肠癌、肺癌、肝癌、胃癌和鼻咽癌^[19]^。值得注意的是，它的表达往往与肿瘤分级和疾病复发呈正相关。研究发现，Chk1高表达的肿瘤细胞更能耐受放疗、化疗或其他肿瘤治疗方法引起的DNA损伤反应，促进产生恶性程度更高的肿瘤细胞，并可导致耐药现象的发生及肿瘤的频繁复发。Nieto等^[20]^研究发现，抑制Chk1可以克服基底样乳腺癌对顺铂的获得性耐药。Fan等^[21]^研究发现，沉默Chk1抑制了慢性髓系白血病（chronic myeloid leukemia, CML）细胞的增殖，且增强了依托泊苷（etoposide, VP16）的细胞毒性作用。其通过降低BRCA1的表达，导致细胞周期阻滞，削弱同源重组（homologous recombination, HR）修复。Nair等^[22]^研究发现一种治疗高级别浆液性卵巢癌的新机制，在Chk1抑制剂的作用下抑制HR，使肿瘤耐药细胞对脱氧核糖核酸损伤剂如吉西他滨或羟基脲敏感，从而导致细胞有丝分裂灾难和细胞死亡。由此可见，抑制Chk1或其抑制剂与产生DNA损伤的药物（如铂化合物、吉西他滨和PARP抑制剂奥拉帕尼）可以在体外对肿瘤迁移、侵袭能力和细胞凋亡有协同作用。靶向DNA修复机制已经在临床得到应用，Chk1抑制剂联合一线抗肿瘤药物，比单独或双联用具有更高的活性，导致更高的凋亡效应。

### 2.2 Chk2与肿瘤耐药

研究^[23-25]^发现，在多种癌症的细胞系中也存在Chk2的改变。Chk2已被证实是一种肿瘤抑制因子，并且在一些癌症中发生突变或减少，包括乳腺癌、结肠癌、膀胱癌、卵巢癌和前列腺癌。Chk2的低表达或缺失对肿瘤有保护作用，可能与肿瘤的发生发展相关。Luo等^[26]^研究发现，在三阴乳腺癌*Chk2* Y390C突变型MDA-MB-231细胞系中，通过激活P53和Chk2-P53凋亡通路，调控细胞凋亡和细胞周期，可以诱导三阴性乳腺癌细胞对化疗药物耐药。Yao等^[27]^研究发现，通过抑制ATM-Chk2-P53信号通路可以促进结直肠癌细胞增殖，抑制细胞凋亡，最终导致5-氟尿嘧啶（5-fluorouracil, 5-FU）耐药。Ta等^[28]^研究发现，前列腺癌患者样本中Chk2的表达下降，认为Chk2是前列腺癌生长的负调控因子，且干扰Chk2信号可能成为治疗前列腺癌的一种新方法。由此可见，Chk2的活化和Chk2的低表达都可能与肿瘤有关，其有助于增加肿瘤的侵袭性，且与部分肿瘤的发生发展、耐药也存在一定的相关性。然而，另有相关研究^[29]^发现，Chk2在食管癌、肝癌、胃癌、高级别浆液性卵巢癌等肿瘤中却存在高表达。因此，对于Chk2和肿瘤之间的调控机制，还需进一步的研究和探讨。

## 3 Chk与肺癌耐药

随着肺癌治疗药物在临床的广泛使用，其耐药性问题越来越突出，已经成为临床治疗的主要障碍。当发生DNA受损时，Chk可通过诱导细胞周期延迟或阻滞，为DNA损伤修复提供时间。近年来，关于Chk表达及其抑制剂与肺癌耐药的研究越来越多，通过解除S期和G_2_期检测点可提高DNA损伤试剂对癌细胞的灵敏度并提高其选择性。有研究^[30]^表明，不仅可以通过应用Chk抑制剂联合化疗或放疗来逆转肺癌耐药，也可以通过单独使用Chk抑制剂终止癌细胞的增殖。

### 3.1 Chk1与肺癌耐药

正常细胞中Chk1的表达极低，而在肺癌组织中，约90%存在Chk1的表达，其基因和蛋白的异常表达能诱导细胞恶性增殖，进而促使肿瘤的进展。研究认为，当Chk1被抑制时癌细胞会失去对DNA损伤的反应和修复能力，从而增强放化疗对细胞的杀伤力。Cai等^[31]^研究发现，F-box蛋白成员Fbxo6，可以通过降低NSCLC中Chk1的表达和磷酸化，抑制细胞增殖，促进细胞凋亡，提高对顺铂（cisplatin, DDP）的敏感性。由此可见，Fbxo6可能是一种有效的治疗靶点，可以克服铂类化疗药物在NSCLC患者中的化疗耐药性。Duan等^[32]^研究发现，一个组蛋白去甲基酶家族JMJD2s，其化学抑制剂使顺铂耐药NSCLC细胞敏感化。抑制JMJD2降低了ATR和Chk1的染色质关联，并抑制了ATR-Chk1复制检测点，结果表明，JMJD2去甲基化酶是克服顺铂耐药的潜在治疗靶点。

与此同时，通过解除Chk1对细胞周期检测点的阻滞作用进而促使肿瘤细胞凋亡，这一机制成为了目前肿瘤研究的新热点，同时也是众多学者寻找潜在的Chk1抑制剂的研究基础。目前已有多种Chk1抑制剂应用于临床，或进入临床试验阶段。

AZD7762是一种强效的Chk1/2双抑制剂，也是一种ATP竞争性药物，能够抑制Chk1介导的Cdc25C磷酸化^[33]^。Grabauskiene等^[34]^研究发现，与低Chk1表达的H1993细胞相比，AZD7762抑制Chk1会优先使高Chk1表达的细胞H1299对抗代谢药物化疗敏感。其对于Chk1蛋白表达水平较高的肿瘤亚群有更强的治疗效果。AZD7762联合化疗药物可降低肿瘤生长，尤其是*p53*突变或缺失的肿瘤。Hsu等^[35]^研究发现，联合AZD7762与DDP可以通过激活caspase-2和下调E2F-1，协同诱导*p53*缺失的小细胞肺癌（small cell lung cancer, SCLC）细胞有丝分裂死亡，抑制Chk1增强了DDP的抗肿瘤活性，并克服了SCLC对DDP的耐药性。

LY2606368（Prexasertib）是一种Chk1/2双重抑制剂，在体外优先结合并抑制Chk1^[36]^。Zhao等^[37]^研究发现在SCLC中，细胞周期调节因子Wee1的DNA拷贝数、mRNA和蛋白水平与细胞耐Prexasertib的程度呈正相关。Wee1 siRNA或Wee1抑制剂可逆转SCLC细胞对Prexasertib的耐药；而Wee1转染可诱导细胞中的Prexasertib耐药性。联合应用Chk1和Wee1抑制剂可能为SCLC的治疗提供一种新的治疗策略。

MK-8776是一种选择性Chk1抑制剂，在异种移植模型中与DNA抗代谢物羟基脲、吉西他滨或培美曲塞联合使用时，可诱导DSBs和细胞死亡^[38]^。Dai等^[39]^研究发现，减少范可尼贫血互补群基因D2（Fanconi anemia complementation group D2, FANCD2）联合MK-8776显著增强了吉西他滨对两株肺癌细胞SK-MES-1和KLN205的细胞毒性。吉西他滨作用的增强伴随着DNA修复功能的丧失和DNA单链断裂和双链断裂的积累，同时caspase-3依赖性凋亡明显增加。

由此可见，抑制Chk1或使用Chk1抑制剂可能在肺癌耐药治疗中发挥着对药物增敏的作用。然而，也有研究表明，Chk1可能并不是肿瘤抑制因子，相反，它促进肿瘤生长，对抗肿瘤药物产生耐药。研究^[40]^还发现过度激活Chk1对肿瘤细胞的生长反而有害，因此认为通过激活而不是抑制Chk1可以达到杀死肿瘤细胞的目的，其可能发展成为癌症治疗的一种创新方法。

### 3.2 Chk2与肺癌耐药

Chk2在肿瘤中的表达水平与肿瘤相关性的研究尚不十分清楚，到目前为止，只有少数研究报道了Chk2在原发性患者组织中的表达。与正常组织相比，Chk2在肺癌中表达缺失或下调，低水平的Chk2在肺癌中被认为是导致化疗耐药的原因^[41]^。Wu等^[42]^研究发现泛素特异肽酶39（ubiquitin specific peptidase39, USP39）是Chk2的一个新的调节分子。它是一种去泛素化酶，可将泛素化的Chk2去泛素化并稳定Chk2，从而提高Chk2的稳定性。此外，敲除USP39可以导致Chk2蛋白水平显著降低，进而调节G_2_期/M期检测点和促进细胞凋亡，从而导致肺癌细胞对化疗药物和放疗的耐药。Luo等^[43]^研究发现，蛋白酶体抑制剂MG132可选择性地上调A549细胞中主要组织相容性复合体I类多肽相关序列B（major histocompatibility complex class I polypeptide related sequence B, MICB）的表达，并增加自然杀伤（natural killer, NK）细胞的细胞毒性。MG132的调控作用可能与Chk2的激活有关，MG132联合NK细胞免疫治疗可能有协同作用，提高肺癌治疗效果。Lin等^[44]^研究发现，11-脱氢水杉内酯是一种具有抗肿瘤生物学特性的软珊瑚化合物，其通过上调P53和Bax，激活ATM和Chk2，激活caspase-3和caspase-7，诱导G_2_期/M期细胞周期阻滞和凋亡，从而抑制Akt通路。由此表明11-脱氢白藜芦醇内酯可能是一种有前途的治疗SCLC的化疗药物。

此外，目前已有学者以Chk2为治疗靶点开发Chk2抑制剂，但研究发现其普遍对Chk1也存在抑制作用，在临床前评估中缺乏特异性。其他临床前数据和早期临床研究表明，Chk1/2双重抑制剂在实体肿瘤中取得了良好的抗肿瘤效果。因此，针对Chk1与Chk2在肺癌的联合研究可能对肺癌耐药的治疗有更重要的意义。

## 4 总结与展望

肺癌是全球最常见的诊断癌症，也是癌症相关死亡的最主要原因。目前临床肿瘤治疗药物均有不同程度的毒副作用，在杀伤肿瘤细胞的同时，对正常的组织细胞也有损伤作用，且肿瘤细胞几乎都会产生耐药性，尤其是多药耐药性（multidrug resistance, MDR）^[45]^。因此，开发针对肺癌耐药作用机制的治疗靶点和治疗方法，逆转肺癌耐药，可以为临床针对肺癌的治疗提供新的策略和思路。

Chk在DDR及细胞周期调控中具有重要作用，与肿瘤的发生发展、复发、耐药及预后相关，可能是一个潜在的抗肿瘤治疗靶点。目前越来越多针对Chk特异性的抑制剂被开发，使得它的作用在临床前环境中得到了广泛的探索。研究证实，Chk的表达及其抑制剂的应用对逆转肺癌耐药有一定的效果，但是能否仅通过调控Chk，从而直接抑制肿瘤的发生和进展尚不是非常的明确。此外，除了与化疗药物联合，Chk抑制剂与其他靶向S期和G_2_期/M期检测点的抑制剂组合能否在肺癌耐药治疗中取得满意疗效，还需进一步探究。虽然Chk抑制剂在化疗增加药物敏感性方面的前景可期，但部分抑制剂的稳定性和安全性还有待进一步的证实。未来，深入了解Chk1和Chk2在肺癌耐药中的作用，将有望提高肺癌的治疗效果，为肺癌患者带来更好的生存及预后。
